# Suppressed pediatric asthma hospitalizations during the COVID‐19 pandemic in Japan, from a national survey

**DOI:** 10.1002/clt2.12330

**Published:** 2024-01-18

**Authors:** Seigo Korematsu, Takao Fujisawa, Naruo Saito, Junichiro Tezuka, Katsushi Miura, Ichiro Kobayashi, Ippei Miyata, Yujiro Kosugi, Yuji Gohda, Yumi Koike, Ami Suda, Akiko Matsuo, Michiyo Sasaki, Yousuke Handa, Michimasa Fujiwara, Atsushi Ono, Shinya Koizumi, Taku Oishi, Takayuki Tanaka, Yusuke Ando, Naohiko Taba, Yuki Tsurinaga, Takeshi Sato, Rei Kanai, Masato Yashiro, Toshiyuki Takagi, Shinya Hida, Masashi Harazaki, Takayuki Hoshina, Seigo Okada, Motoko Yasutomi, Setsuko Nakata, Ayako Muto, Saori Tanabe, Yutaka Ueda, Shunji Hasegawa, Makoto Kameda, Keiko Tanaka‐Taya, Tsuguto Fujimoto, Kenji Okada

**Affiliations:** ^1^ Department of Pediatrics Saitama Medical Center Saitama Medical University Saitama Japan; ^2^ Allergy Center NHO Mie National Hospital Mie Japan; ^3^ Saito Clinic for Asthma and Allergy Children Shiga Japan; ^4^ Department of Allergy and Pulmonology Fukuoka Children's Hospital Fukuoka Japan; ^5^ Department of Allergy Miyagi Children's Hospital Miyagi Japan; ^6^ Center for Pediatric Allergy and Rheumatology KKR Sapporo Medical Center Sapporo Japan; ^7^ Department of Pediatrics Kawasaki Medical School Okayama Japan; ^8^ Department of Pediatrics Nakatsu Municipal Hospital Oita Japan; ^9^ Department of Pediatrics Sasebo Kyosai Hospital Nagasaki Japan; ^10^ Department of Allergy Nagano Children's Hospital Nagano Japan; ^11^ Department of Pediatrics Ageo Central General Hospital Saitama Japan; ^12^ Department of Pediatrics Yokohama City Minato Red Cross Hospital Kanagawa Japan; ^13^ Department of Pediatrics Nagasaki University Nagasaki Japan; ^14^ Department of Pediatrics Kitsuki City Yamaga Hospital Oita Japan; ^15^ Department of Pediatrics NHO Fukuyama Medical Center Hiroshima Japan; ^16^ Department of Pediatrics Hiroshima City Funairi Citizens Hospital Hiroshima Japan; ^17^ Department of Pediatrics The Fraternity Memorial Hospital Tokyo Japan; ^18^ Department of Pediatrics Kochi Medical School Kochi University Kochi Japan; ^19^ Department of Pediatrics Japanese Red Cross Otsu Hospital Shiga Japan; ^20^ Department of Pediatrics Dokkyo Medical University Tochigi Japan; ^21^ Department of Pediatrics Fukuoka National Hospital Fukuoka Japan; ^22^ Department of Pediatrics Osaka Habikino Medical Center Osaka Japan; ^23^ Department of Pediatrics Soka Municipal Hospital Saitama Japan; ^24^ Department of Pediatrics Okayama University Okayama Japan; ^25^ Department of Pediatrics Showa University Tokyo Japan; ^26^ Department of Pediatrics Osaka Red Cross Hospital Osaka Japan; ^27^ Department of Pediatrics Shizuoka General Hospital Shizuoka Japan; ^28^ Department of Pediatrics School of Medicine University of Occupational and Environmental Health Fukuoka Japan; ^29^ Department of Pediatrics Yamaguchi University Graduate School of Medicine Yamaguchi Japan; ^30^ Department of Pediatrics Faculty of Medical Sciences University of Fukui Fukui Japan; ^31^ Department of Pediatrics Matsumoto City Hospital Nagano Japan; ^32^ Department of Pediatrics Tokyo Women's Medical University Yachiyo Medical Center Chiba Japan; ^33^ Department of Pediatrics Nihonkai Hospital Yamagata Japan; ^34^ Department of Pediatrics Saitama Medical University Hospital Saitama Japan; ^35^ Kanagawa Prefectural Institute of Public Health Kanagawa Japan; ^36^ Department of Fungal Infection National Institute of Infectious Diseases Tokyo Japan; ^37^ Department of Basic Nursing Fukuoka Nursing College Fukuoka Japan

**Keywords:** bronchial asthma, COVID‐19, enterovirus D68, national survey, pediatrics

## Abstract

**Background:**

Acute asthma exacerbation in children is often caused by respiratory infections. In this study, a coordinated national surveillance system for acute asthma hospitalizations and causative respiratory infections was established. We herein report recent trends in pediatric acute asthma hospitalizations since the COVID‐19 pandemic in Japan.

**Methods:**

Thirty‐three sentinel hospitals in Japan registered all of their hospitalized pediatric asthma patients and their causal pathogens. The changes in acute asthma hospitalization in children before and after the onset of the COVID‐19 pandemic and whether or not COVID‐19 caused acute asthma exacerbation were investigated.

**Results:**

From fiscal years 2010–2019, the median number of acute asthma hospitalizations per year was 3524 (2462–4570), but in fiscal years 2020, 2021, and 2022, the numbers were 820, 1,001, and 1,026, respectively (the fiscal year in Japan is April to March). This decrease was observed in all age groups with the exception of the 3‐ to 6‐year group. SARS‐CoV‐2 was evaluated in 2094 patients from fiscal years 2020–2022, but the first positive case was not detected until February 2022. Since then, only 36 of them have been identified with SARS‐CoV‐2, none of which required mechanical ventilation. Influenza, RS virus, and human metapneumovirus infections also decreased in FY 2020. In contrast, 24% of patients had not been receiving long‐term control medications before admission despite the severity of bronchial asthma.

**Conclusion:**

SARS‐CoV‐2 was hardly detected in children with acute asthma hospitalization during the COVID‐19 pandemic. This result indicated that SARS‐CoV‐2 did not induce acute asthma exacerbation in children. Rather, infection control measures implemented against the pandemic may have consequently reduced other respiratory virus infections and thus acute asthma hospitalizations during this period. However, the fact that many hospitalized patients have not been receiving appropriate long‐term control medications is a major problem that should be addressed.

## 1 INTRODUCTION

The outbreak of the coronavirus infectious disease 2019 (COVID‐19) caused by severe acute respiratory syndrome coronavirus‐2 (SARS‐CoV‐2) began in Wuhan in December 2019, and the World Health Organization announced a global pandemic in January 2020.^1^

Bronchial asthma in children can be acutely exacerbated by respiratory infectious agents such as rhinovirus (RV), respiratory syncytial virus (RSV), human metapneumovirus (hMpV), and influenza virus.^2^ This prompted concerns that bronchial asthma might be acutely exacerbated by SARS‐CoV‐2.^3^ However, based on eight epidemiological reports, Matsumoto et al.^4^ found that the prevalence of bronchial asthma in more than 17,000 patients with COVID‐19 (average 5.3%) was lower than the general prevalence in their region (average 8.0%). Patients with severe COVID‐19 also had a significantly higher prevalence of chronic obstructive pulmonary disease and diabetes than non‐severe patients (both *p* = 0.000), but the prevalence of bronchial asthma was not significantly different (*p* = 0.111).

In 2014, there was an enterovirus D68 (EV‐D68) epidemic in a wide area of the world, mainly in the United States, and in September 2015, it also became an epidemic in Japan. During this epidemic, acute asthma exacerbation, acute respiratory failure, and acute flaccid paralysis cases increased, and EV‐D68 was detected in some cases, suggesting that this infection was the cause. A nationwide retrospective survey of acute asthma hospitalizations of children was carried out for the period from January 2010 through October 2015. The Japanese Society of Pediatric Allergy and Clinical Immunology asked its affiliated hospitals to report monthly numbers of hospitalizations, intensive‐care unit admissions, and mechanical ventilation due to acute asthma exacerbation. A total of 157 hospitals reported 87,189 asthma hospitalizations, including 477 intensive‐care unit admissions and 1193 mechanical ventilation, during the survey period of 5 years and 10 months. The number of these events increased drastically in September 2015.^5^

This experience has led us to establish a coordinated national surveillance system for acute asthma hospitalizations and their causative respiratory infections to promote measures against acute asthma exacerbation in Japan. Since April 2020, 33 sentinel hospitals have been prospectively monitored for acute asthma exacerbation in children. In addition, we also investigated pathogens detected in children hospitalized with acute asthma exacerbation.

In this study, we surveyed changes in acute asthma hospitalization in children after the onset of the COVID‐19 pandemic and investigated whether or not COVID‐19 causes acute asthma exacerbation.

## 2 MATERIALS AND METHODS

The Japan Pediatric Asthma Exacerbation and Respiratory Infections (JPAERI) Registry is a nationwide registry of pediatric asthma hospitalizations in Japan that registers acute asthma hospitalization simultaneously with its respiratory pathogens assumed to cause. The JPAERI registry aims to determine the domestic incidence of acute asthma exacerbation requiring hospitalization in patients under 19 years of age and to determine associations of these events with respiratory pathogens. This registry operates in real‐time, rather than retrospectively, allowing for early detection of outbreaks of respiratory infections that cause asthma exacerbation and consideration of early intervention.

**FIGURE 1:**
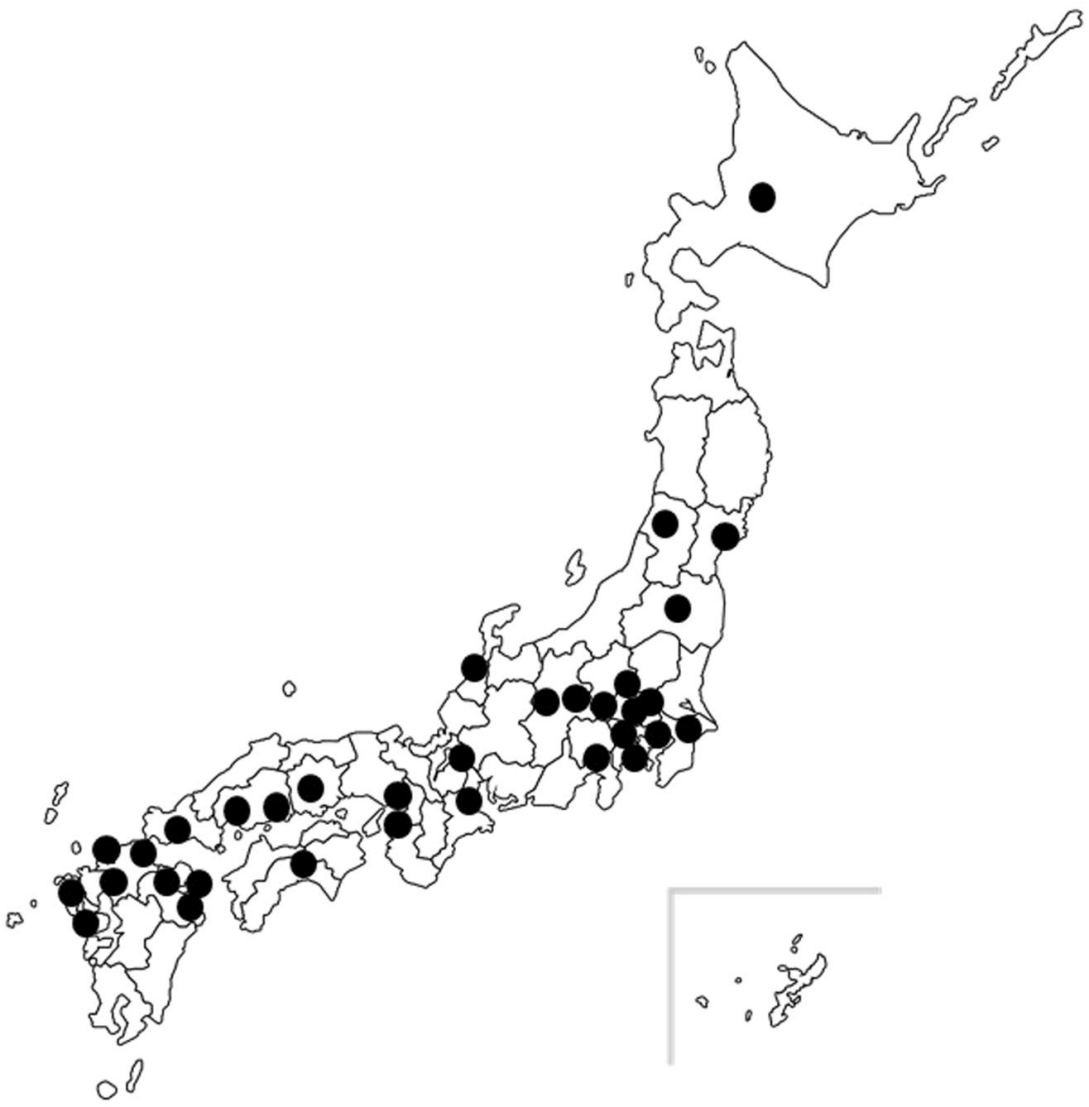
Sentinel hospitals participating in this study. The 33 sentinel hospitals in Japan are indicated by black circles.

The Japanese Society of Pediatric Allergy and Clinical Immunology and the Japanese Society of Pediatric Infectious Diseases recruited 33 widely distributed sentinel hospitals in 2022 (Figure 1). Pediatricians of these hospitals registered cases of pediatric acute asthma hospitalization on the website using two predefined criteria: 1) a diagnosis of asthma or asthmatic bronchitis, and 2) hospital admission. Patients requiring admission and/or mechanical ventilation, including nasal high flow and continuous positive airway pressure, were also registered. The registry is open to the public, and the above‐mentioned data after 2010 can be browsed by facility, prefecture, region, nationwide, and even by age group.

In this registry, we retrospectively entered the age, sex, and availability of mechanical ventilation for children hospitalized for acute asthma exacerbation from fiscal year (FY) 2010–2022 (from April 2010 to March 2023. The FY in Japan is April to March). From FY 2020, we prospectively added data with pathogens detected in respiratory specimens of children hospitalized with acute asthma exacerbation. As pathogen examinations were routinely performed as health insurance coverage tests at each facility and registered, not all hospitalized patients were tested for a pathogen using a unified protocol. Furthermore, the details of methods for detecting pathogens such as antigen and PCR tests were not registered. From FY 2020, we added data on asthma severity and long‐term control medications being taken as of admission. The severity criteria were based on the Japanese Pediatric Asthma Guidelines.^6^ This guideline recommends long‐term control medications from the mild persistent type, mainly leukotriene receptor antagonists and/or inhaled corticosteroids.

Furthermore, we also examined the number of hospitalizations due to respiratory infections, namely influenza, RSV, and hMpV, at sentinel hospitals from FY 2020 to 2022.

Due to the research funding period, each year's totals were based on the FY of funding. Thus, retrospective data were collected beginning in April 2010, and the registry began in April 2022.

Written informed consent from patients and guardians was obtained, and a website with additional information and an opt‐out button was set up for the study. This research was performed using a grant from the Ministry of Education, Culture, Sports, Science, and Technology in Japan, and conducted under ethical approval by the Saitama Medical University, Saitama Medical Center (approval number: SMC2021‐037).

## 3 RESULTS

1. Trends in acute asthma‐hospitalized patients

**FIGURE 2:**
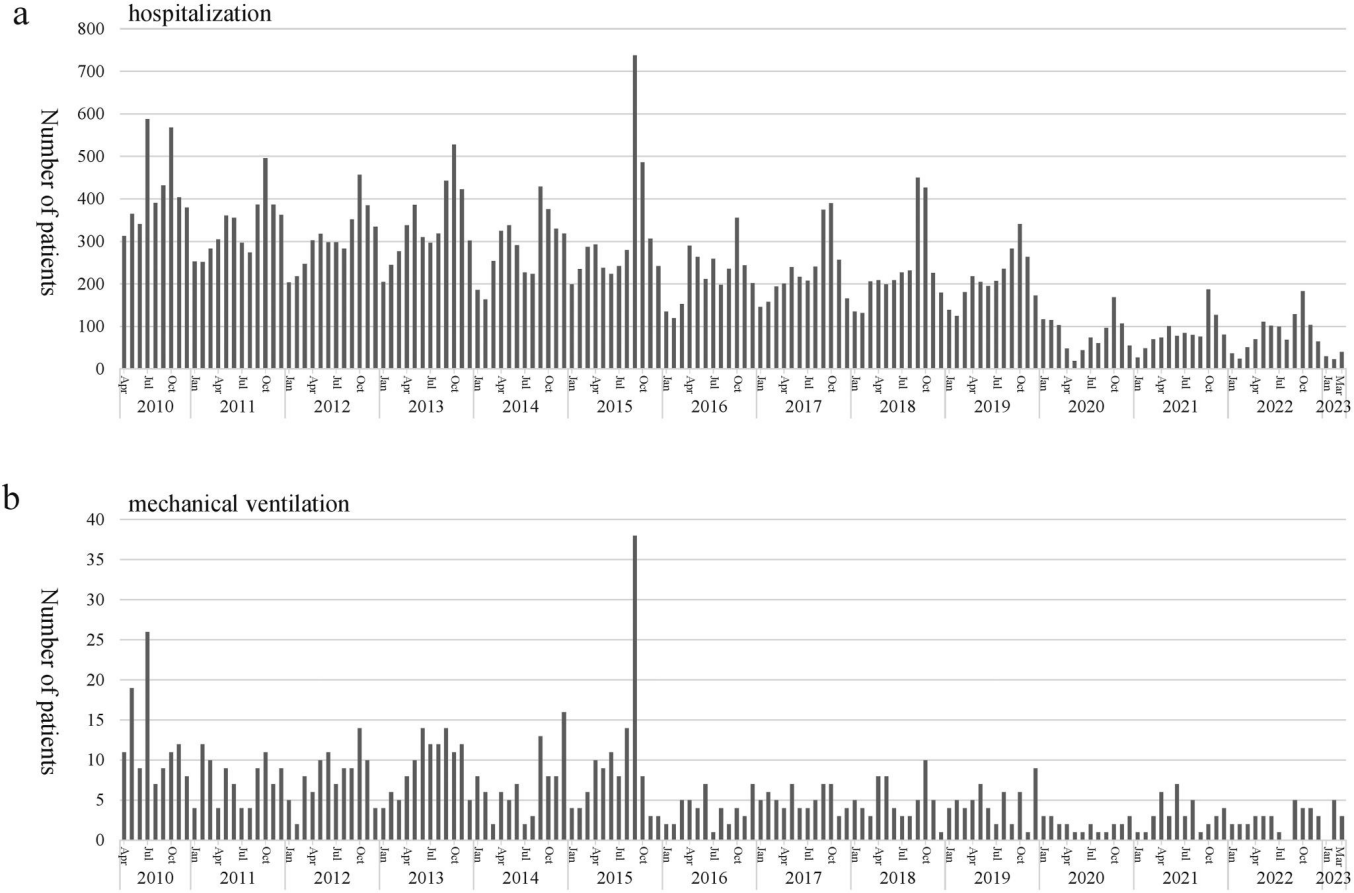
Trends in hospitalization for acute asthma. (A) Acute asthma hospitalization and (B) mechanical ventilation. Both acute asthma hospitalization and patients requiring mechanical ventilation decreased after the COVID‐19 pandemic.

**FIGURE 3:**
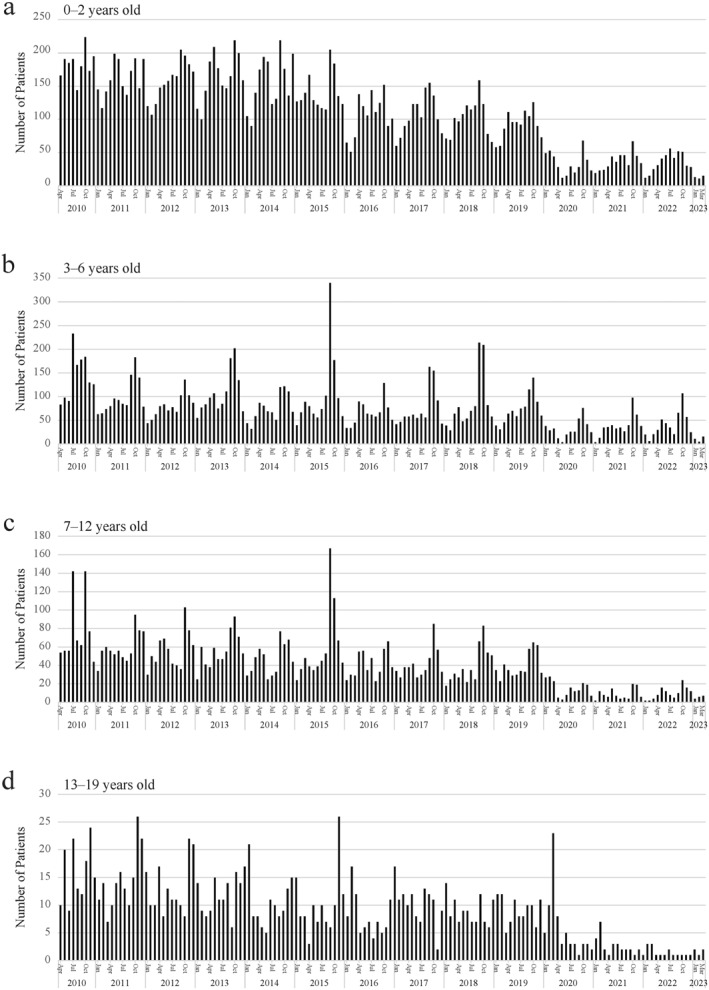
Trends in acute asthma hospitalizations by age group. (A), 0–2 years; (B), 3–6 years; (C), 7–12 years, (D); 13–19 years.

From FY 2010 to 2019, the median number of acute asthma hospitalizations per year was 3524 (2462–4570), but in FY 2020, 2021, and 2022, the numbers were 820, 1,001, and 1,026, respectively (Figure 2A). Similarly, from FY 2010 to 2019, the number of patients requiring mechanical ventilation per year was 80.5 (53–138), but in FY 2020, 2021, and 2022, those numbers were 20,40, and 34, respectively (Figure 2B). During this research period, September 2015 was the point when the number of acute asthma hospitalizations was the highest and the number of cases of mechanical ventilation management increased. It was in this very month that EV‐D68 caused an epidemic. Figure 3 shows the age group‐stratified trends in acute asthma hospitalizations. A decrease during the pandemic was observed in all age groups and the reduction was most evident in FY2020. For the 0–2 and 3–6 age groups, however, there was a slight increase in asthma hospitalizations in FY2021 and 2022 although the numbers were still less than those before the pandemic.

2. Detection of SARS‐CoV‐2 and other pathogens

**FIGURE 4:**
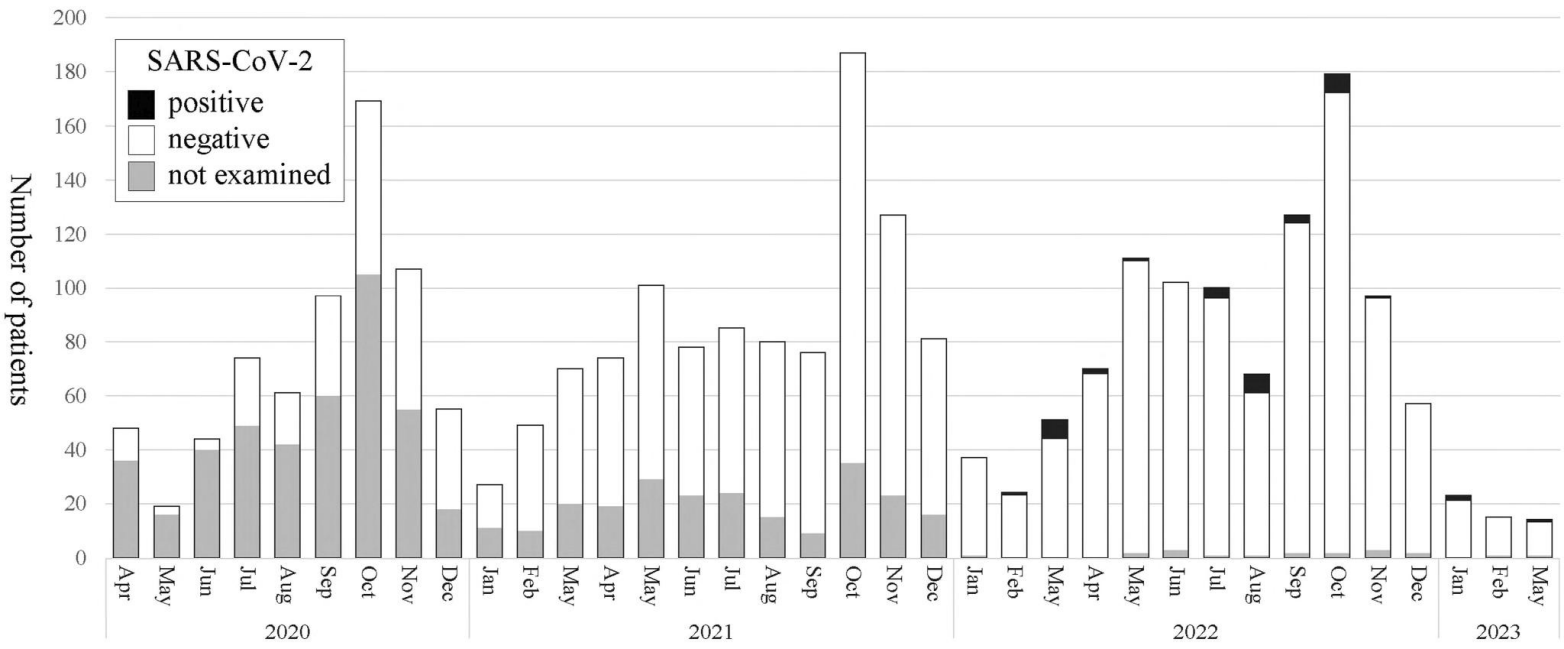
Detection of SARS‐CoV‐2. Black, SARS‐CoV‐2 positive; white, SARS‐CoV‐2 negative; gray, not tested. Patients hospitalized for acute asthma exacerbation were rarely positive for SARS‐CoV‐2.

SARS‐CoV‐2 was evaluated in 2094 patients from FY 2020–2022, but the first positive case was not detected until February 2022. Since then, a total of 36 SARS‐CoV‐2‐positive cases have been identified (Figure 4), none of which required mechanical ventilation.

**FIGURE 5:**
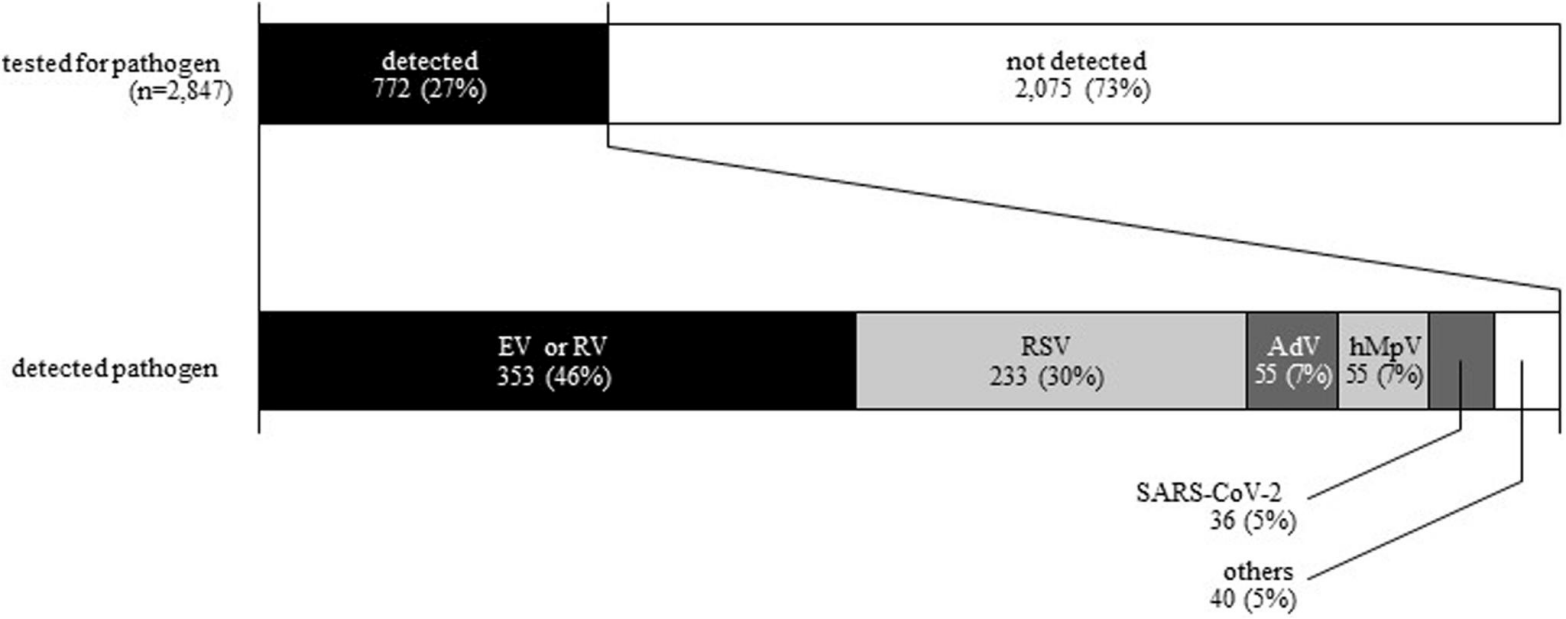
Detection of pathogens among acute asthma hospitalized patients from FY 2020–2022. EV, enterovirus; hMpV, human metapneumovirus; RV, rhinovirus; RSV, respiratory syncytial virus; SARS‐CoV‐2, severe acute respiratory syndrome coronavirus‐2. The top bar shows the pathogen detection rate and the bottom bar shows the pathogens detected.

Among acute asthma hospitalizations from FY 2020–2022, pathogens were detected in 772 patients (27%). The breakdown was RV or enterovirus (EV) in 353 patients (46%), RSV in 233 patients (30%), adenovirus in 55 patients (7%), hMpV in 55 patients (7%), SARS‐CoV‐2 in 36 patients (5%), and others in 40 patients (5%) (Figure 5). A total of 86 patients required mechanical ventilation, and their causative pathogens were RV or EV in 21 and RSV in 12.

3. Severity of bronchial asthma and long‐term control medications before admission

The severity of bronchial asthma among hospitalized patients from FY 2020–2022 was initial onset in 730, intermittent type in 840, mild persistent type in 519, moderate persistent type in 474, severe persistent type in 167, and most severe persistent type in 24, while details were unknown for 195. Twenty‐four percent (282/1184) of patients with mild persistent type (165/519), moderate persistent type (110/474), severe persistent type (7/167), and most severe persistent type (0/24) were untreated (Table 1) despite their bronchial asthma severity requiring long‐term control medications before admission, according to the guideline.

4. Trends of influenza, RSV, and hMpV infection in hospitalized patients

**TABLE 1:** The severity of bronchial asthma and long‐term control medications before admission.

Severity	Total	Long‐term control medication		
		Inhaled steroid and/or leukotriene receptor antagonist	Neither inhaled steroid nor leukotriene receptor antagonist	Others
Initial onset	730	7	723	3
Intermittent	840	132	708	5
Mild persistent	519	354	165	8
Moderate persistent	474	364	110	18
Severe persistent	167	160	7	30
Most severe persistent	24	24	0	10
Detail unknown	195	124	71	1
Total	2949	1165	1784	75

Since respiratory viral infection is a major risk factor for exacerbation of asthma, we additionally surveyed the number of children hospitalized due to respiratory infections with influenza, RSV, and hMpV at the sentinel hospitals from FY 2014–2022. We found that the hospitalization related to all three pathogens drastically decreased in FY2020, although RSV infections returned to the pre‐pandemic level in FY2021, and influenza and hMpV infections returned to the pre‐pandemic level in FY2022 (Table 2).

**TABLE 2:** Number of patients hospitalized with viral infection.

	Influenza	RSV infection	hMpV infection
FY 2014	401	1404	165
FY 2015	600	1482	99
FY 2016	428	1333	275
FY 2017	610	1797	319
FY 2018	506	1337	363
FY 2019	643	1465	457
FY 2020	18	158	35
FY 2021	33	1567	11
FY 2022	283	1122	445

Abbreviations: FY, fiscal year; hMpV, human metapneumovirus; RSV, respiratory syncytial virus.

## 4 DISCUSSION

This study investigated the effect of the COVID‐19 pandemic based on changes in pediatric acute asthma hospitalizations in Japan since 2010. During this research period, an extraordinarily large spike in pediatric acute asthma hospitalizations was observed in September 2015, which was found to be associated with the EV‐D68 epidemic. In contrast, acute asthma hospitalizations decreased after the onset of the COVID‐19 pandemic. SARS‐CoV‐2 was evaluated in 1602 patients from April 2020 to October 2022, but the first positive case was not detected until February 2022. Since then, a total of 36 SARS‐CoV‐2‐positive patients have been identified, none of which required mechanical ventilation. The results of this national survey in Japan indicated that SARS‐CoV‐2 played a minor role in acute asthma exacerbation in children. Infection control measures that have been implemented against the pandemic may have reduced other respiratory virus infections, but 24% of hospitalized patients were untreated despite their bronchial asthma severity requiring long‐term control medications before admission.

In the early stages of the COVID‐19 pandemic, there was concern that it might cause acute asthma exacerbation, similar to RV, RSV, influenza virus, and EV‐D68.^2^, ^5^ However, Sunjaya et al.^7^ conducted a meta‐analysis of 51 papers from December 2019 to July 2021 and reported that the odds ratio of contracting COVID‐19 in patients with bronchial asthma was 0.83 (0.73–0.95), which is lower than in patients without bronchial asthma. In the United States, Ye et al.^8^ also investigated average weekly bronchial asthma emergency department visits and reported a 31% decline relative to what was observed during the comparison period (from 45,276 visits/week in 2019 to 31,374 visits/week in 2020). Declines of over 19% and 26% were observed for 2021 and 2022, respectively. Shar et al.^9^ analyzed the United Kingdom primary care database from 2016 and pointed out that the acute asthma exacerbation rate in 100,362 patients from the first quarter of 2016 to the first quarter of 2020 ranged from 48.7 (44.6–52.8) to 88.9 (83.4–94.4), while it decreased from 23.0 (21.6–24.4) to 33.8 (31.5–36.2) from the second quarter of 2020 to the third quarter of 2021.

In pediatric patients, Du et al.^10^ reported the clinical course of 182 children with COVID‐19 in Wuhan and found no marked difference in COVID‐19 severity compared with non‐allergic patients. In contrast, they also reported that procalcitonin, D‐dimer, and AST showed less marked elevation in allergic children. In a systematic review of 67 studies up to May 2020, Castro‐Rodriguez et al.^11^ and Choi et al.^12^ reported no evidence that pediatric bronchial asthma is a risk factor for developing severe COVID‐19. Abe et al.^13^ also reported that the number of acute asthma hospitalizations under 18 years old decreased after the start of the COVID‐19 pandemic in Japan, using a national database of Diagnosis Procedure Combinations collected since 2017. Kivistö et al.^14^ also found that the incidence of pediatric asthma hospitalizations decreased by 36.7% in Finland and by 39.9% in Sweden from 2015 to 2019. However, these reports describe trends before the emergence of the Omicron variant, when the number of pediatric COVID‐19 patients was small. Our report suggested that there were similar trends after the emergence of the Omicron variant, which increased pediatric COVID‐19.

SARS‐CoV‐2 is a variant of the human coronavirus. Even before the COVID‐19 pandemic, the pooled prevalence (95% CI) during acute asthma exacerbation in children was 45.7% (37.5%–53.8%) for RV, 17.7% (13.2%–23.7%) for RSV, and 11.8% for EV (6.2%–22.5%), while that of human coronavirus was only 8.4% (5.1%–13.6%).^2^ As the pooled prevalence of human coronaviruses in adults was 20.8% (12.0%–36.1%),^2^ it is suggested that children may be less likely than adults to have acute asthma exacerbation due to human coronavirus infection. Abe et al.^15^ also reported that from 2012 to 2015, human coronavirus counted for only 2% of pathogens detected in nasopharyngeal swabs from 175 of 216 hospitalized children with bronchial asthma.

Several reports have focused on the angiotensin‐converting enzyme 2 (ACE2) as a reason why COVID‐19 is not a risk factor in bronchial asthma and children. SARS‐CoV‐2 uses ACE2 as a receptor in human cells. Bunyavanich et al.^16^ investigated ACE2 levels in the nasal mucosa of 305 individuals aged 4–60 years and reported that its expression was age‐dependent and significantly lower in children than in adults. Jackson et al.^17^ reported that the expression of ACE2 in the airway epithelium of patients with bronchial asthma was significantly lower than that of healthy subjects. Kimura et al.^18^ reported that IL‐13, a type 2 helper T‐cell cytokine that is an important biomarker in the pathogenesis of bronchial asthma, reduces the expression of ACE2 in airway epithelial cells. Furthermore, Jackson et al.^17^ described ACE2 expression in airway epithelial cells as being downregulated when allergic patients were exposed to antigens.

Why did acute asthma exacerbations decrease during the pandemic? There are several possible explanations. First, lower rates of patient consultations and hospital admissions may have affected the decrease. There was certainly a time in the early days of the pandemic when people avoided going to the hospital out of fear of contracting COVID‐19. However, it is very unlikely that patients with severe asthma symptoms would not seek medical care since emergency pediatric departments continued to accept severely ill children during the pandemic in Japan.

Second, the asthma treatment status may have changed sufficiently to reduce exacerbations, but the survey regularly conducted by the Japanese Society of Pediatric Allergy and Clinical Immunology showed that the use of inhaled corticosteroids and other asthma controllers was similar in 2014, 2016, and 2020.,^19^ which denies the possibility of treatment change.

Instead, the decline in other respiratory infections due to strengthened infection control measures during the pandemic is a likely explanation for the decline in acute asthma exacerbations since respiratory virus infection is the major trigger of asthma exacerbation.^20^ To support this idea, we separately surveyed trends in overall hospitalizations due to three major respiratory virus infections at our sentinel hospitals. In fact, in FY 2020, hospitalization due to influenza, RSV, and hMpV infections greatly reduced to about one‐10th of the previous rate, which appeared to coincide precisely with the reduction in asthma hospitalizations.

However, hospitalizations due to RSV infections and other infections returned to pre‐pandemic levels in FY2021 and FY2022, respectively, while the number of asthma hospitalizations stayed at a low level during the same period. We believe this discrepancy can be explained as follows. Respiratory virus infections act on asthma not only as direct triggers of exacerbation^20^ but also as promoters of asthma inception. It has been reported that RSV infection in early life leads to type 2 immune responses causing allergen sensitization and airway inflammation.^21^ Early RSV‐associated lower respiratory tract infections^22^ and any respiratory infections^23^ are significant risk factors for later development of recurrent wheezing and asthma. Thus, the protection of young children from respiratory viral infection in the first year of the pandemic may have protected them from the development of airway inflammation and asthma and resulted in low exacerbation rates in the second and third years of the pandemic. Further long‐term follow‐up will test the hypothesis.

On the other hand, the fact that 24% of patients had not been receiving long‐term control medications before admission despite their bronchial asthma severity is a major problem that should be addressed. According to the Japanese guideline, long‐term control medications are recommended from the mild persistent type, mainly leukotriene receptor antagonists and inhaled corticosteroids.^5^ Because this surveillance system did not obtain these data before the COVID‐19 pandemic, this phenomenon is not understood whether due to avoiding hospital visits during the COVID‐19 pandemic or not. To prevent and cure pediatric asthma, it is important not only to prevent infectious diseases but also to control asthmatic patients daily.

Several limitations associated with the present study warrant mention. First, only 33 sentinel hospitals were involved, and because input was requested from the doctors in charge of each facility, the input contents could not be verified, and there might also be a time lag in the input. Second, pathogen tests are not performed in all patients, as they are only performed based on the judgment of the attending physician, and in many patients, only those approved under the scope of Japanese medical insurance. For example, RSV antigen can only be evaluated in children under 1 year old, children with underlying diseases for which palivizumab is indicated, and children who are hospitalized. Similarly, the hMpV antigen can only be tested for children under 6 years old. Following the spread of COVID‐19, multiplex polymerase chain reaction tests of respiratory pathogens for severe illness patients became covered by the Japanese insurance system, but not all sentinel hospitals carried out this test. Furthermore, because this survey does not include pathogen data before 2020, causative pathogen trends in acute asthma exacerbation before the COVID‐19 pandemic are unclear. Finally, because this survey did not collect information about SARS‐CoV‐2 vaccination, the effect of the SARS‐CoV‐2 vaccine on acute asthma exacerbation has not been elucidated. The indications of its vaccination have been gradually expanded to children (e.g., children≥12 years old from June 2021, children 5–11 years old from March 2022, and children 6 months to 4 years old from October 2022).

No report has demonstrated a decline in pediatric asthma patients during the COVID‐19 pandemic using national data from more than 10 years ago, and no report has demonstrated a low rate of detection of SARS‐CoV‐2 in patients hospitalized with acute asthma exacerbation. We believe that continuing this approach will further clarify the relationship between bronchial asthma and respiratory infections.

## AUTHOR CONTRIBUTIONS

**Seigo Korematsu**: Conceptualization (lead); data curation (lead); formal analysis (lead); funding acquisition (lead); investigation (lead); methodology (lead); project administration (lead); resources (lead); software (lead); supervision (lead); validation (lead); visualization (lead); writing—original draft (lead); writing—review and editing (equal). **Takao Fujisawa**: Conceptualization (equal); investigation (equal); supervision (equal); writing—review and editing (equal). **Naruo Saito**: Software (equal); writing—review and editing (equal). **Junichiro Tezuka**: Data curation (equal); investigation (equal); writing—review and editing (equal). **Katsushi Miura**: Data curation (equal); investigation (equal); writing—review and editing (equal). **Ichiro Kobayashi**: Data curation (equal); investigation (equal); writing—review and editing (equal). **Ippei Miyata**: Investigation (equal); visualization (supporting); writing—review and editing (equal). **Yujiro Kosugi**: Data curation (equal); investigation (equal); writing—review and editing (equal). **Yuji Gohda**: Data curation (equal); investigation (equal); writing—review and editing (equal). **Yumi Koike**: Data curation (equal); investigation (equal); writing—review and editing (equal). **Ami Suda**: Data curation (equal); investigation (equal); writing—review and editing (equal). **Akiko Matsuo**: Data curation (equal); investigation (equal); writing—review and editing (equal). **Michiyo Sasaki**: Data curation (equal); investigation (equal); writing—review and editing (equal). **Yousuke Handa**: Data curation (equal); investigation (equal); writing—review and editing (equal). **Michimasa Fujiwara**: Data curation (equal); investigation (equal); writing—review and editing (equal). **Atsushi Ono**: Data curation (equal); investigation (equal); writing—review and editing (equal). **Shinya Koizumi**: Data curation (equal); investigation (equal); writing—review and editing (equal). **Taku Oishi**: Data curation (equal); investigation (equal); writing—review and editing (equal). **Takayuki Tanaka**: Data curation (equal); investigation (equal); writing—review and editing (equal). **Yusuke Ando**: Data curation (equal); investigation (equal); writing—review and editing (equal). **Naohiko Taba**: Data curation (equal); investigation (equal); writing—review and editing (equal). **Yuki Tsurinaga**: Data curation (equal); investigation (equal); writing—review and editing (equal). **Takeshi Sato**: Data curation (equal); investigation (equal); writing—review and editing (equal). **Rei Kanai**: Data curation (equal); investigation (equal); writing—review and editing (equal). **Masato Yashiro**: Data curation (equal); investigation (equal); writing—review and editing (equal). **Toshiyuki Takagi**: Data curation (equal); investigation (equal); writing—review and editing (equal). **Shinya Hida**: Data curation (equal); investigation (equal); writing—review and editing (equal). **Masashi Harazaki**: Data curation (equal); investigation (equal); writing—review and editing (equal). **Takayuki Hoshina**: Data curation (equal); investigation (equal); writing—review and editing (equal). **Seigo Okada**: Data curation (equal); investigation (equal); writing—review and editing (equal). **Motoko Yasutomi**: Data curation (equal); investigation (equal); writing—review and editing (equal). **Setsuko Nakata**: Data curation (equal); investigation (equal); writing—review and editing (equal). **Ayako Muto**: Data curation (equal); investigation (equal); writing—review and editing (equal). **Saori Tanabe**: Data curation (equal); investigation (equal); writing—review and editing (equal). **Yutaka Ueda**: Data curation (equal); investigation (equal); writing—review and editing (equal). **Shunji Hasegawa**: Supervision (equal); writing—review and editing (equal). **Makoto Kameda**: Supervision (equal); writing—review and editing (equal). **Keiko Tanaka‐Taya**: Supervision (equal); writing—review and editing (equal). **Tsuguto Fujimoto**: Supervision (equal); writing—review and editing (equal). **Kenji Okada**: Supervision (equal); writing—review and editing (equal).

## CONFLICT OF INTEREST STATEMENT

The authors declare no conflicts of interest.

## KEY MESSAGE

During the COVID‐19 pandemic, children with acute asthma hospitalization decreased, and SARS‐CoV‐2 was hardly detected in these children. SARS‐CoV‐2 did not induce acute asthma exacerbation in children.

## Data Availability

Our data are available in the https://asthma‐attack.children.jp/view.php?page=index.

## References

[1] Wu D , Wu T , Liu Q , Yang Z . The SARS‐CoV‐2 outbreak: what we know. Int J Infect Dis. 2020;94:44‐48. 10.1016/j.ijid.2020.03.004 32171952 PMC7102543

[2] Zheng XY , Xu YJ , Guen WJ , Lin LF . Regional, age and respiratory‐secretion‐specific prevalence of respiratory viruses associated with asthma exacerbation: a literature review. Arch Virol. 2018;163(4):845‐853. 10.1007/s00705-017-3700-y 29327237 PMC7087223

[3] Cianferoni A , Votto M . COVID‐19 and allergy: how to take care of allergic patients during a pandemic? Pediatr Allergy Immunol. 2020;31(Suppl 26):96‐101. 10.1111/pai.13367 33236431 PMC7753363

[4] Matsumoto K , Saito H . Does asthma affect morbidity or severity of COVID‐19? J Allergy Clin Immunol. 2020;46(1):55‐57. 10.1016/j.jaci.2020.05.017 PMC7250068 32470485

[5] Korematsu S , Nagashima K , Sato Y , et al. “Spike” in acute asthma exacerbations during enterovirus D68 epidemic in Japan: a nationwide survey. Allergol Int. 2018;67(1):55‐60. 10.1016/j.alit.2017.04.003 28455155

[6] Arakawa H , Adachi Y , Ebisawa M , et al. Committee for Japanese pediatric guideline for childhood asthma; Japanese society of pediatric allergy and clinical Immunology, and Japanese society of allergology. Japanese guidelines for childhood asthma 2020. Allergol Int. 2020;69(3):314‐330. 10.1016/j.alit.2020.02.005 33213779

[7] Sunjava AP , Allida SM , Di Tanna GL , Jenkins CR . Asthma and COVID‐19 risk: a systematic review and meta‐analysis. Eur Respir J. 2022;59(3):2101209. 10.1183/13993003.01209-2021 34385278 PMC8361304

[8] Ye D , Gates A , Radhakrishnan L , Mirabelli MC , Flanders WD , Sircar K . Changes in asthma emergency department visits in the United States during the COVID‐19 pandemic. J Asthma. 2023;60(8):1601‐1607. 10.1080/02770903.2023.2165445 36608267 PMC10293019

[9] Shar SA , Quint JK , Sheikh A . Impact of COVID‐19 pandemic on asthma exacerbations: a retrospective cohort study of over 500,000 patients in a national English primary care database. Lancet Regional Health. 2022;19:100428. 10.1016/j.lanepe.2022.100428 PMC9213032 35756853

[10] Du H , Dong X , Zhang JJ , et al. Clinical characteristics of 182 pediatric COVID‐19 patients with different severities and allergic statuses. Allergy. 2021;76(2):510‐532. 10.1111/all.14452 32524611 PMC7307120

[11] Castro‐Rodriguez JA , Forno E . Asthma and COVID‐19 in children: a systematic review and call for data. Pediatr Pulmonol. 2020;55(9):2412‐2418. 10.1002/ppul.24909 32558360 PMC7323291

[12] Choi JH , Choi SH , Yun KW . Risk factor for severe COVID‐19 in children: a systematic review and meta‐analysis. J Kor Med Sci. 2022;37(5):e35. 10.3346/jkms.2022.37.e35 PMC8822112 35132841

[13] Abe K , Miyawaki A , Nakamura M , Ninomiya H , Kobayashi Y . Trends in hospitalizations for asthma during the COVID‐19 outbreak in Japan. J Allergy Clin Immunol Pract. 2021;9(1):494‐496. 10.1016/j.jaip.2020.09.060 33065368 PMC7553873

[14] Kivistö JE , Protudjer JLP , Karjalainen J , et al. Paediatric asthma hospitalizations continue to decrease in Finland and Sweden between 2015 and 2020. Thorax. 2023;78(3):313‐315. thorax‐2022‐219539 Online ahead of print. 10.1136/thorax-2022-219539 36593115

[15] Abe N , Yasudo H , Fukano R , et al. Multi‐season analyses of causative pathogens in children hospitalized with asthma exacerbation. Pediatr Allergy Immunol. 2019;30(7):724‐731. 10.1111/pai.13102 31251831 PMC7167852

[16] Bunyavanich S , Do A , Vicencio A . Nasal gene expression of angiotensin‐converting enzyme 2 in children and adults. JAMA. 2020;323(23):2427‐2429. 10.1001/jama.2020.8707 32432657 PMC7240631

[17] Jackson DJ , Busse WW , Bacharier LB , et al. Association of respiratory allergy, asthma, and expression of the SARS‐CoV‐2 receptor ACE2. J Allergy Clin Immunol. 2020;46(1):203‐206. 10.1016/j.jaci.2020.04.009 PMC7175851 32333915

[18] Kimura H , Francisco D , Conway M , et al. Type 2 inflammation modulates ACE2 and TMPRSS in airway epithelial cells. J Allergy Clin Immunol. 2020;146(1):80‐88. 10.1016/j.jaci.2020.05.004 32422146 PMC7227558

[19] Itazawa T , Odajima H , Iino A , et al. A multicenter study of changes in asthma severity distribution over time. 2020 report. Jpn J Ped Allergy Clin Immunol. 2022;36:119‐126. In Japanese.

[20] Coverstone AM , Wang L , Sumino K . Beyond respiratory syncytial virus and rhinovirus in the pathogenesis and exacerbation of asthma: the role of metapneumovirus, bocavirus and influenza virus. Immunol Allergy Clin. 2019;39(3):391‐401. 10.1016/j.iac.2019.03.007 PMC7127190 31284928

[21] Krishnamoorthy N , Khare A , Oriss TB , et al. Early infection with respiratory syncytial virus impairs regulatory T cell function and increases susceptibility to allergic asthma. Nat Med. 2012;18(10):1525‐1530. 10.1038/nm.2896 22961107 PMC3641779

[22] Shi T , Ooi Y , Zaw EM , et al. Association between respiratory syncytial virus‐associated acute lower respiratory infection in early life and recurrent wheeze and asthma in later childhood. J Infect Dis. 2020;222(Supplment_7):S628‐S633. 10.1093/infdis/jiz311 31370064

[23] Nagasaki T , Tabuchi T , Matsumoto H , Horimukai K . Age‐specific associations of early daycare, older siblings, severe airway infection, and preterm birth with subsequent atopic diseases. Pediatr Allergy Immunol. 2022;33(4):e13771. 10.1111/pai.13771 35470939

